# Mapping amorphous SiO_2_ in Devonian shales and the possible link to marine productivity during incipient forest diversification

**DOI:** 10.1038/s41598-023-28542-y

**Published:** 2023-01-27

**Authors:** H. Corlett, J. Feng, T. Playter, B. Rivard

**Affiliations:** 1 Department of Earth Sciences, Memorial University of Newfoundland and Labrador, St. John’s, NL A1B 3X5 Canada; 2 Department of Earth and Atmospheric Sciences, University of Alberta, 1-26 Earth Sciences Building, Edmonton, AB T6G 2E3 Canada; 3 Alberta Geological Survey, Alberta Energy Regulator, Edmonton Regional Office 402, Twin Atria Building, 4999 - 98 Avenue, Edmonton, AB T6B 2X3 Canada

**Keywords:** Biogeochemistry, Climate sciences, Ecology, Ocean sciences

## Abstract

Silica cycling in the world’s oceans is not straightforward to evaluate on a geological time scale. With the rise of radiolarians and sponges from the early Cambrian onward, silica can have two depositional origins, continental weathering, and biogenic silica. It is critical to have a reliable method of differentiating amorphous silica and crystalline silica to truly understand biogeochemical and inorganic silica cycling. In this study, opal-A is mapped across the Western Canada Sedimentary Basin in the Late Devonian Duvernay Formation shales using longwave hyperspectral imaging alongside geochemical proxies that differentiate between crystalline and amorphous SiO_2_, during the expansion of the world’s early forests. Signaled by several carbon isotope excursions in the Frasnian, the *punctata* Event corresponds to the expansion of forests when vascular land plants develop seeds and deeper root networks, likely resulting in increased pedogenesis. Nutrients from thicker soil horizons entering the marine realm are linked to higher levels of primary productivity in oceans and subsequent oxygen starvation in deeper waters at this time. The results of this study reveal, for the first time, the spatial distribution of amorphous SiO_2_ across a sedimentary basin during this major shift in the terrestrial realm when forests expand and develop deeper root networks.

## Introduction

A major shift in climate and oxygen levels in Earth’s atmosphere beginning near the Emsian-Eifelian boundary (~ 395 Ma)^1^ and continued into the Early Frasnian when forests were expanding^2–4^. The world’s first forests were identified in the late Emsian in Spitzbergen and in Givetian strata in Gilboa, New York, USA^5,6^, however, Capel et al.^3^ identifies several major origination-extinction pulses during the Silurian-Devonian that eventually resulted in a transition to a forested terrestrial landscape during the Middle Devonian. By the end of the Givetian, root networks had deepened and by the Frasnian, aneurophyte and archaeopterid progymnosperm forests were common, resulting in thicker soil horizons starting to form; thereby increasing terrestrially-derived nutrient delivery to the marine environment^2,4,7^. Previous studies of these shifts in biodiversity predicted that enhanced nutrient delivery may have caused increases in productivity, oxygen stratification, deposition of organic rich black shales, and eutrophication in Frasnian epicontinental seas^2,4,8,9^. Middle to Late Devonian lacustrine sediments from Greenland and northern Scotland reveal a net loss in phosphorous (P), an essential biolimiting nutrient that is expected to decrease in a terrestrial environment that is undergoing plant colonization where P is liberated from minerals indirectly through the acidification of root pore spaces produced by the degradation of organic matter and release of organic exudates from roots^8,10,11^. A significant and long-lasting δ^13^C shift in the *punctata* conodont zone, thought to be caused by the increased delivery of liberated nutrients (e.g. P) that would enhance productivity and burial of organic carbon in the Middle to Late Devonian, is referred to *punctata* Event (pE) and is recognized in basins worldwide^12^. Suspected productivity associated with the pE may also result in amplification of biologically sourced amorphous SiO_2_ in areas that experienced nutrient influx through the delivery of soils formed by deeper root networks^2,8^. Amorphous SiO_2_ has been consistently underestimated in ancient sedimentary sequences, which distorts our understanding of global biogeochemical silica cycling^13–16^. Silica in shales was commonly interpreted as terrigenous in origin; however, Schieber^17^ and Schieber et al.^14^ demonstrated that significant proportions of quartz silt in shales could be biogenically- or diagenetically-derived, especially after the early Cambrian when radiolarians and siliceous sponges started to proliferate^16^. The SiO_2_-rich Frasnian Duvernay Formation shales displays δ^13^C_(org)_ excursions characteristic of the pE, which have also been documented in the Canadian Rocky Mountains^18^. These basinal deposits are therefore examined in this study to determine whether SiO_2_ in the Duvernay shales is of biological origin and whether increases in SiO_2_ deposition could be linked to the significant shift in the terrestrial realm when the world’s forests were expanding.

Differentiation of SiO_2_ polymorphs is possible but is challenging on a macroscale. Currently the methods used to differentiate amorphous (possibly biogenic) versus crystalline SiO_2_ are:

1. Identification of siliceous microfossils or SiO_2_-filled cysts^17,19^, textures recognized in petrographic work such as irregularly shaped grains with embayments and pointy projections^17,20^, pyrite inclusions^17,19^, and quartz grains with colloform or chalcedonic textures^14,17^;
2. Scanning electron microscope cathodoluminescence imaging or energy dispersive x-ray spectroscopy^14,17,19,21,22^;
3. Oxygen isotope values^14,23^;
4. Silica excess; defined by Rowe et al.^24^ as the absolute value of the difference between the measured silica content and the silica versus aluminium regression line, which represents silica in the aluminosilicate phase (i.e., clay minerals)^20^;
5. A negative correlation between silica and zirconium^25^;
6. A positive correlation between silica and TOC^20^;
7. X-ray diffraction, where peak height differences are used as a crystallinity index^26^
8. Alkaline digestion^27^.

Each of these methodologies requires spot analysis where a specific interval is targeted through sampling. To detect SiO_2_ on a larger scale, our study uses longwave infrared spectroscopy (LWIR) in the 8–12 µm wavelength range^28^. LWIR can differentiate between amorphous and crystalline SiO_2_ due to asymmetric stretching of Si–O–Si bonds in amorphous SiO_2_^29,30^. Using this method of detection can enhance our understanding of biogeochemical SiO_2_ cycling through time, since it enables in situ detection on the macroscale, thereby allowing us to map amorphous SiO_2_ distributions in ancient sedimentary basins and track shifts in bioproductivity that may coincide with significant climatic shifts.

Three drill cores through the Duvernay shales from different locations across the Western Canada Sedimentary Basin in Alberta were sampled at regularly spaced intervals for whole-rock geochemistry and stable isotope analyses. Shales are less susceptible than carbonate rocks to diagenetic alteration that may affect δ^13^C, used to identify the pE. Each core is analysed for δ^13^C_(org)_ to determine if the pE excursions are recorded in each of these locations. SiO_2_ provenance is investigated using oxide data and geochemical proxies to determine whether there is any “excess silica”^24^ present in the Duvernay and if so, what the source may be. Any SiO_2_ that is contributed from a biological source, signalling bioproductivity associated with the pE, would likely be an amorphous polymorph of SiO_2_. To detect and map amorphous SiO_2_, the cores are imaged using a longwave infrared (LWIR) drill core hyperspectral scanner. The aims of the study are to determine whether the pE is recorded at each location in the basin, to identify intervals of amorphous SiO_2_, and to determine the source of SiO_2_ using several geochemical proxies and hyperspectral imaging. Possible sources of amorphous SiO_2_ include hydrothermal fluids, SiO_2_ produced as a by-product of clay diagenesis, or biogenic SiO_2_. A biogenic source of SiO_2_ in the Duvernay, deposited during expansion of the world’s forests, would support current theories that the development of deeper *Archaeopteris* tree roots resulted in increased soil genesis and riverine nutrient delivery^8^, ultimately increasing bioproductivity and deposition of organic material in the oceans at this time^2,4,12^. This study, focused on differentiation and mapping of SiO_2_ polymorphs contributes to our understanding of silica in the world’s oceans through time, which is linked to global carbon, oxygen and climate cycles^27^. The novel technique used to differentiate detrital versus amorphous SiO_2_ may provide a critical advancement in our ability to quantify biogenic SiO_2_ in sediment, which is considered a high priority in developing a better understanding of biogeochemical silica cycling^8,27^.

### Geological background

**Figure 1:**
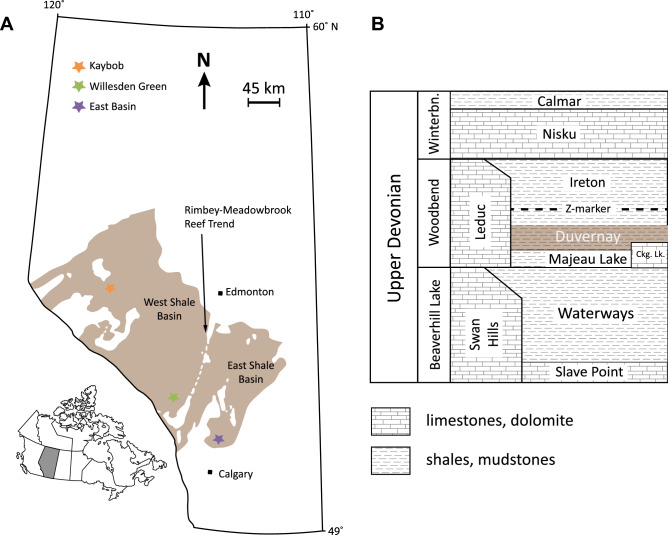
(**A**) Map of Alberta with extent of the subsurface Duvernay Formation. modified from the Geological Atlas of the Western Canada Sedimentary Basin^28^. (**B**) Simplified stratigraphy of the Duvernay Formation in Alberta, Canada and the location of three drill cores used in this study.

The Duvernay Formation shales are organic-rich, calcareous to argillaceous/siliceous shales that were deposited during the Frasnian-age (382–372 Ma) Woodbend Group (Fig. 1), when the WCSB was a passive margin on the western passive margin of the North American craton^31^. The most up to date conodont stratigraphy from the WCSB places the lower portion of the Duvernay in the *punctata* conodont zone (Montagne Noire (MN) 5/6) and the upper Duvernay mostly in the *hassi* conodont zone (MN7-10)^31^. During this time, present-day Alberta was covered by a large interior seaway, with numerous carbonate buildups that grew in succession. The Leduc Formation formed on paleotopographic highs on the thickest portions of the underlying Swan Hills Formation platform in the west, and the Cooking Lake Formation platform in the east^32,33^. The first two stages of reef growth in the Leduc Formation are coeval with deposition of the Duvernay basinal mudstones and shales^33^. In the east, the Duvernay directly overlies the Cooking Lake Formation platform carbonates and in the west, the basinal equivalent Majeau Lake Formation. These western and eastern portions of the WCSB, known as the West Shale Basin and East Shale Basin respectively, are separated by the Rimbey-Meadowbrook reef trend (Fig. 1)^34^.

The Duvernay is highly heterogeneous both locally and across its 130,000 km^2^ depositional extent^35^. The Duvernay, or the Perdrix as it is referred to in the Rocky Mountain exposures, is comprised of ten lithofacies, defined by composition, grain size, and sedimentary structures in Knapp et al.^36^. Broadly, the Duvernay is composed of organic rich siliceous mudstones, carbonate, and clay-rich shales^36^. Based on informal litho- and chemostratigraphy and areas of production from the Duvernay, it is divided into three domains herein referred to as: Kaybob, Willesden Green (WG), and East Shale Basin (ESB) (Fig. 1). In the Kaybob area, the Duvernay has seven geochemically distinct units, and is characterized by the presence of a middle carbonate unit^37,38^. In our study, the Kaybob well is divided into the upper Duvernay, middle Duvernay (carbonate-rich), and the lower Duvernay. In the Willesden Green (WG) area, the Duvernay is comprised of thin, intercalated shale and fine-grained carbonate beds. In the East Shale Basin (ESB), the Duvernay is surrounded by the Leduc Formation reefs and as a result is relatively carbonate rich throughout, in comparison to the WG and the upper and lower Duvernay in the Kaybob well.

## Analytical methods

Three Duvernay Formation cores were sampled and imaged in this study; 100/04-19-064-22W5/00 (Kaybob), 100/09-25-039-06W5/00 (Willesden Green = WG), and 100/08-29-031-23W4/00 (East Shale Basin = ESB; Fig. 1).

### Data collection

A total of 303 samples were taken at 0.5–1 m intervals from three Duvernay drill cores. The samples were analysed for major elements using inductively coupled plasma optical emission spectrometry (ICP-OES). Samples from the Kaybob drill core were analysed for elemental chemistry using an iCAP 7000 Series ICP-OES (Thermo Fisher Scientific, Waltham, MA, USA) at Chemostrat Inc. laboratories in Houston, Texas, following the procedure outlined in Hildred et al.^39^. Samples from the Willesden Green and East Shale Basin cores were analysed using a Spectro ICP-OES (Perkin Elmer, Waltham, MA, USA) at Bureau Veritas Mineral Laboratories in Vancouver, British Columbia. Oxides analysed in the samples include: SiO_2_, TiO_2_, Al_2_O_3_, Fe_2_O_3_, MgO, MnO, CaO, Na_2_O, K_2_O, and P_2_O_5_. Total organic carbon (TOC) and δ^13^C_(org)_ (reported relative to VPDB) were analysed at Chemostrat Inc. for the Kaybob well, and at the University of Saskatchewan Stable Isotope Laboratory for the WG and ESB. All of the samples analysed for δ^13^C_(org)_ and TOC were acidified to remove carbonate material, homogenized, and the organic material oxidized to carbon dioxide, nitrogen bearing gases, and water, which are further separated for analysis. Isotopes were measured at Chemostrat Inc. using a Europa Scientific 2020 Isotope Ratio Mass Spectrometer and at the Saskatchewan Stable Isotope Laboratory using a Thermo Finnigan Flash 1112 EA coupled to a Thermo Finnigan Delta Plus XL through a Conflo III, where they were calibrated against international standards L-SVEC (δ^13^C_(org)_ = − 46.6 VPDB) and IAEA-CH-6 (δ^13^C_(org)_ = − 10.45 VPDB) with a lab reported precision of 0.12% (n = 18, 2σ). Fifty polished thin sections were prepared and examined from the Kaybob well. Each section was impregnated with blue epoxy to highlight pore space, and the sections were half stained with Alizarin red to recognize calcite and aragonite. The thin sections were examined using a Zeiss Axioscope A1 at MacEwan University in Edmonton, Alberta. Twenty-five samples from the Kaybob well were also analysed for mineral content using X-ray Diffraction (XRD) at the Chemostrat Inc. laboratories in Houston, Texas.

### Data analysis

SiO_2_ and Al_2_O_3_ data were used to identify the presence of excess silica that is not considered to have been derived from continental crust, using Eq. (1)^20^.

1 $${\text{oxide}}_{{{\text{EX}}}} = {\text{oxide}}_{{{\text{sample}}}} {-}\left[ {\left( {{\text{oxide}}/{\text{Al}}_{2} {\text{O}}_{3} } \right)_{{{\text{background}}}} \times {\text{Al}}_{2} {\text{O}}_{{3\;{\text{sample}}}} } \right]$$

A value of 3.527 was used for average shale or background^40^. The amount of excess silica (Si_EX_) is reported alongside δ^13^C_(org)_ data. The most recently reported conodont biostratigraphy presented in Wong et al.^31^ was used to correlate δ^13^C_(org)_ data collected in the Duvernay with previous studies of the *punctata* Event^12^. Data analysis was performed on oxides and TOC analysed in the three wells using multivariate statistics (Principal Component Analysis; PCA) in DataDesk® 6.3.1. to determine SiO_2_ provenance. PCA analyzes the total variance of the oxide and TOC dataset to determine the relationship of SiO_2_ to clay-associated oxides (e.g. Al_2_O_3_, TiO_2_, K_2_O, MgO, Fe_2_O_3_, Na_2_O), carbonates (e.g. CaO, MnO), or to variables that represent a biological influence (e.g. P_2_O_5_, TOC)^41^. In this study, eigen vectors e1 versus e2 and e1 versus e3 were plotted for each well to account for greater than 85% of variance in the dataset.

A potential hydrothermal source for amorphous SiO2 is evaluated using an Al–Fe-Mn ternary plot. Adachi et al.^42^ defines a zone that implies a hydrothermal influence near the Fe-apex (up to 30% Mn) and non-hydrothermal as being Al-rich, and Mn-poor. Samples from each well in this study are plotted on this ternary diagram.

Clay diagenesis (e.g. K-metasomatism) can produce amorphous SiO_2_ as a by-product^43^. To determine whether the clays in the Duvernay have undergone significant diagenetic alteration that may have introduced amorphous SiO_2_, oxide data is used to first determine the chemical index of alteration (CIA), which assesses the degree of weathering that the sediments have undergone from their source (Eq. 2)^44,45^.

2 $${\text{CIA}} =_{{{\text{molar}}}} \left[ {\left( {{\text{Al}}_{2} {\text{O}}_{3} } \right)/\left( {{\text{Al}}_{2} {\text{O}}_{3} + {\text{CaO}}* + {\text{ Na}}_{2} {\text{O}} + {\text{K}}_{2} {\text{O}}} \right)} \right] \, \times 100$$

CaO* should represent Ca only in the silicate portion^44,45^. Following McLennan^44^, CaO was corrected for using phosphate data where CaO* is equal to the moles of CaO minus moles of P_2_O_5_ × 10/3. This value is compared to the moles of Na_2_O, and if the corrected value of CaO* is less than moles of Na_2_O, than this CaO* value is used in Eq. 2, otherwise CaO* is equal to Na_2_O.

CIA values calculated for sediments that have experienced diagenesis through K-metasomatism need to be corrected using an A-CN-K (Al_2_O_3_–CaO* + Na_2_O–K_2_O) plot where molar values of Al_2_O_3_, (CaO* + Na_2_O), and K_2_O are plotted on a ternary diagram. A typical weathering trend for sediment parallels the A-CN line, where the sediment loses Ca, Na, and K as it becomes more weathered from the original source rock. The level of K-enrichment, reflecting K-metasomatism and possible amorphous silica by-product, can be estimated by projecting each sample plotted on the A-CN-K ternary diagram back to the assumed, pre-metasomatized, original position (CIA_corr_) along the weathering trend line (A-CN parallel line)^16,46–48^. The level of diagenesis experienced in each well is calculated by determining the difference between CIA and CIA_corr_ for each sample using the A-CN-K plot. Samples from each well were plotted on an A-CN-K plot. For the Kaybob well, XRD data was used to determine which samples should be included, since the A-CN-K plot is only used for analyzing weathering and K-enrichment in siliciclastic sediments. Therefore, only the lower (3304.36–3333.25 m) and upper Duvernay (3346.15–3359.13 m) were included in the analysis and the middle Duvernay (3333.25–3346.15 m) samples were excluded as these are almost entirely composed of carbonate.

### Spectral imaging

**Figure 2:**
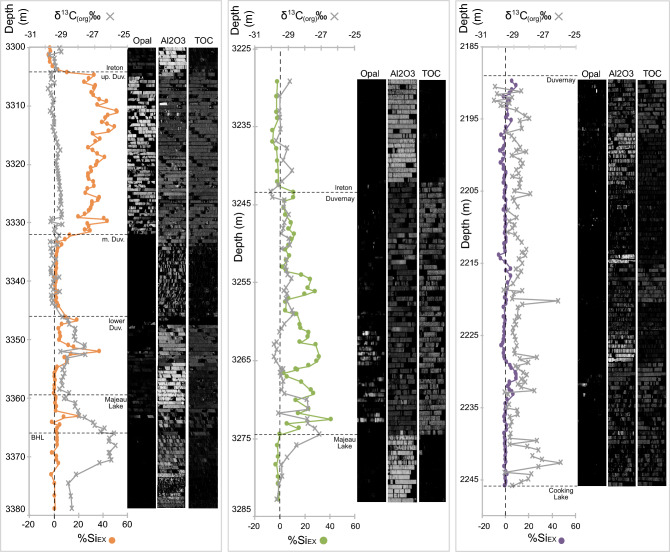
Si_EX_, and δ^13^C_(org)_ plotted versus depth; LWIR hyperspectral images of detected opal-A, and predicted Al_2_O_3_, and TOC (LWIR; greyscale). The dashed line refers to 0% Si_EX_. Left, middle, and right panels correspond to Kabob, Willesden Green and East Shale Basin respectively. The greyscale images have minimum and maximum image line values for opal-A index, %Al_2_O_3_, and %TOC of: 0.0–5.2, 0.0–19.5 (15.2), 0.0–4.2 (4.35) (Kaybob); 0.0–5.4, 0.0–15.2 (15.9), 0.0–5.5 (13.2) (Willesden Green); 0.0–4.3, 0.0–18.3(11.4), 0.0–4.1 (9.8) (East Shale Basin).

Short- (SWIR; 970–2510 nm) and longwave (LWIR; 7400–12,100 nm) infrared reflectance spectra of the three Duvernay cores were collected at the University of Alberta using imaging spectrometers part of commercial SisuROCK systems. Cores boxes, containing slabbed Duvernay cores marked with intervals of spot samples taken for geochemistry, were scanned at a spatial resolution of 0.8 (SWIR) and 0.85 (LWIR) mm/pixel. We predicted TOC and Al_2_O_3_ in core imagery using spectral models developed for shales as detailed in Rivard et al.^28^ The models are based on spectral attributes (e.g., absorption features) related to mineralogy or TOC. The development of the models involved four steps: (1) matching core intervals sampled for geochemistry and TOC to corresponding hyperspectral imagery to generate a representative reflectance spectrum per sample interval, (2) wavelet analysis to highlight mineralogical features in reflectance spectra, (3) use of correlation scalograms to select key spectral features predictive of TOC and geochemistry, and (4) regression analysis to generate predictive models. A predicted weight percent value (TOC and Al_2_O_3_) is computed for each pixel and results for the core are displayed as greyscale images (Fig. 2). We also estimate the relative abundance of opal-A in core imagery. Amorphous SiO_2_ is readily distinguished from crystalline SiO_2_ in the Duvernay where amorphous SiO_2_ has a single reflectance peak near 9000 nm and crystalline SiO_2_ has a double reflectance peak near 8400 and 9200 nm^28^. The difference in the reflectance spectra is caused by Si–O–Si stretching in crystalline quartz as compared to amorphous quartz^30,49^. An opal-A index was designed to measure the relative strength of the two SiO_2_ features in the spectra of each image pixels and compare changes across the image. The strength of the features was measured from continuum removed spectra. Specifically, the opal-A index was computed as (CRa-CRb)/(CRa + CRb) where CRa and CRb are respectively the average continuum removed emissivity from 8312–8493 nm and 9017–9206 nm. The carbonate-rich and clay-rich pixels were excluded for this calculation. The index value is computed for each pixel and displayed as a greyscale image for the core. (Fig. 2).

## Results

All oxide, δ^13^C_(org)_, and TOC values for the three wells analysed in this study are detailed in Table 1.

**Table 1:** δ^13^C_(org)_ reported as ‰ VPDB, total organic carbon (TOC), and oxide data for the Kaybob (K), Willesden Green (WG), and East Shale Basin (ESB) wells.

Well	Depth (m)	TOC	δ^13^C_(org)_	Al_2_O_3_	SiO_2_	TiO_2_	Fe_2_O_3_	MnO	MgO	CaO	Na_2_O	K_2_O	P_2_O_5_
K	3304.36	2.637	− 29.677	12.197	49.108	0.625	4.769	0.033	2.884	8.024	0.499	3.726	0.182
K	3304.82	2.650	− 29.627	8.636	59.885	.446	3.256	0.023	1.713	5.804	0.477	2.494	0.115
K	3305.50	2.898	− 29.657	9.682	58.985	0.483	3.830	0.022	1.780	6.531	0.488	2.870	0.159
K	3306.10	1.090	− 29.417	4.801	40.009	0.288	3.937	0.076	3.775	20.486	0.431	1.437	0.072
K	3306.65	2.494	− 29.673	8.004	59.127	0.403	3.136	0.023	1.774	7.043	0.436	2.305	0.106
K	3307.10	2.935	− 29.835	8.039	58.296	0.398	3.564	0.026	1.906	7.670	0.387	2.349	0.101
K	3307.85	2.977	− 29.675	7.601	54.789	0.379	3.274	0.025	1.805	8.712	0.374	2.243	0.130
K	3308.30	2.993	− 29.632	7.841	56.887	0.390	3.336	0.027	1.865	8.319	0.391	2.316	0.138
K	3309.00	2.896	− 29.463	5.847	55.823	0.354	3.802	0.031	2.661	8.325	0.447	1.710	0.121
K	3309.35	2.505	− 29.527	5.269	59.928	0.310	3.183	0.030	2.372	8.406	0.431	1.519	0.127
K	3309.97	2.782	− 29.542	7.565	59.168	0.385	3.376	0.018	1.418	6.600	0.482	2.181	0.156
K	3310.90	1.880	− 29.702	3.096	61.287	0.165	1.572	0.017	0.922	7.433	0.351	0.835	0.100
K	3311.15	2.211	− 29.591	4.051	64.622	0.213	2.028	0.017	1.038	6.508	0.387	1.113	0.116
K	3311.70	2.297	− 29.527	5.347	60.810	0.296	2.337	0.023	1.346	8.296	0.426	1.524	0.139
K	3312.20	2.820	− 29.461	6.315	61.966	0.328	2.574	0.018	1.210	6.746	0.391	1.862	0.119
K	3312.85	2.971	− 29.465	6.430	59.776	0.328	3.053	0.019	1.134	7.886	0.380	1.850	0.120
K	3313.20	2.705	− 29.467	5.677	61.591	0.293	2.694	0.019	1.107	7.588	0.370	1.627	0.109
K	3313.70	2.931	− 29.565	5.235	65.919	0.270	2.496	0.017	1.022	6.753	0.395	1.499	0.109
K	3314.40	2.719	− 29.509	6.294	64.692	0.334	2.842	0.017	1.131	6.106	0.453	1.792	0.125
K	3314.90	3.058	− 29.405	8.435	58.144	0.420	6.626	0.018	1.499	6.375	0.456	2.543	0.165
K	3315.80	3.544	− 29.366	6.526	58.225	0.327	2.991	0.018	1.261	7.585	0.407	1.936	0.136
K	3316.25	3.650	− 29.283	8.252	59.766	0.402	3.029	0.018	1.250	7.787	0.445	2.355	0.162
K	3317.18	3.235	− 29.195	9.001	56.167	0.425	3.579	0.023	1.431	9.795	0.507	2.574	0.163
K	3317.65	2.972	− 29.084	8.301	57.789	0.382	2.646	0.022	1.342	8.477	0.477	2.379	0.139
K	3318.30	2.412	− 29.252	9.570	65.757	0.410	3.152	0.017	1.548	3.781	0.532	2.740	0.126
K	3318.85	2.099	− 29.252	8.380	67.713	0.368	2.972	0.021	1.630	4.516	0.505	2.357	0.124
K	3319.65	2.289	− 29.325	9.355	64.915	0.418	3.113	0.020	1.545	5.467	0.492	2.710	0.113
K	3320.25	2.337	− 29.295	9.804	65.372	0.419	3.425	0.020	1.605	4.359	0.521	2.817	0.127
K	3320.65	2.418	− 29.285	11.234	65.351	0.485	3.822	0.020	1.819	3.420	0.595	3.221	0.119
K	3321.20	2.152	− 29.249	10.829	64.399	0.411	3.207	0.021	1.583	4.163	0.533	3.077	0.131
K	3321.75	2.190	− 29.219	11.816	65.149	0.427	3.481	0.018	1.670	2.529	0.590	3.386	0.102
K	3322.15	2.175	− 29.220	11.037	62.331	0.426	3.594	0.018	1.660	2.687	0.553	3.185	0.104
K	3322.60	2.268	− 29.173	10.211	60.770	0.403	3.172	0.018	1.601	2.409	0.532	2.939	0.094
K	3323.10	2.545	− 29.135	10.663	65.706	0.459	3.312	0.018	1.676	2.973	0.546	3.198	0.110
K	3323.80	2.761	− 29.117	10.057	64.551	0.432	3.217	0.017	1.565	3.972	0.492	3.071	0.126
K	3324.23	2.394	− 29.145	12.490	66.395	0.433	3.214	0.016	1.552	2.306	0.620	3.755	0.102
K	3324.95	2.733	− 29.047	11.888	62.858	0.453	3.256	0.019	1.753	3.162	0.636	3.574	0.093
K	3325.80	4.355	− 29.054	7.204	59.948	0.355	3.020	0.020	1.356	9.176	0.471	2.259	0.138
K	3326.25	3.419	− 29.073	8.707	63.172	0.414	2.798	0.018	1.445	6.821	0.483	2.768	0.169
K	3326.50	3.959	− 29.075	8.374	57.435	0.447	3.981	0.029	1.954	9.105	0.416	2.497	0.115
K	3326.87	4.142	− 29.022	8.017	56.208	0.391	3.214	0.019	1.387	9.649	0.482	2.525	0.158
K	3327.60	2.926	− 29.182	7.449	53.806	0.365	5.277	0.018	1.285	7.565	0.454	2.365	0.169
K	3328.05	3.452	− 29.033	9.480	58.479	0.486	3.512	0.016	1.390	5.611	0.504	3.043	0.145
K	3328.55	3.085	− 28.981	9.534	55.357	0.453	3.415	0.016	1.407	5.522	0.497	3.082	0.142
K	3329.05	4.481	− 29.027	10.850	53.887	0.534	3.866	0.019	1.702	7.884	0.553	3.436	0.182
K	3329.40	2.532	− 29.612	5.538	58.590	0.281	2.615	0.017	1.000	8.670	0.415	1.752	0.111
K	3329.80	2.902	− 29.695	6.445	64.003	0.358	2.731	0.016	1.068	6.545	0.432	2.024	0.126
K	3330.30	3.076	− 28.969	11.314	63.081	0.510	3.572	0.015	1.726	2.432	0.561	3.554	0.120
K	3330.70	1.728	− 29.378	4.024	41.298	0.167	3.650	0.035	0.965	23.590	0.469	1.194	0.312
K	3331.20	3.389	− 29.470	10.530	58.543	0.538	4.291	0.019	1.764	6.839	0.545	3.330	0.194
K	3331.45	2.832	− 29.405	10.687	63.112	0.472	3.880	0.016	1.659	4.381	0.509	3.333	0.123
K	3332.20	0.749	− 29.141	5.279	29.604	0.229	1.809	0.035	1.469	31.079	0.384	1.569	0.072
K	3332.85	1.582	− 29.298	7.495	32.762	0.299	2.559	0.027	1.885	24.961	0.479	2.246	0.071
K	3333.25	0.155	− 29.707	1.215	6.135	0.049	0.672	0.027	0.774	47.588	0.188	0.308	0.034
K	3333.75	0.391	− 29.242	5.212	23.061	0.216	1.989	0.041	1.591	34.433	0.389	1.492	0.053
K	3334.40	0.489	− 29.682	5.191	19.698	0.215	1.930	0.038	1.715	35.634	0.365	1.502	0.073
K	3335.05	0.524	− 29.599	6.194	22.019	0.270	2.191	0.035	1.869	33.210	0.276	1.887	0.070
K	3335.65	0.459	− 29.608	6.550	22.898	0.276	2.252	0.036	1.944	32.858	0.280	1.979	0.069
K	3336.30	0.138	− 29.339	1.403	5.999	0.052	0.583	0.026	0.883	48.370	0.154	0.390	0.032
K	3337.05	0.446	− 29.485	4.346	16.059	0.190	1.608	0.034	1.441	39.781	0.195	1.300	0.057
K	3337.85	0.318	− 29.674	4.548	16.509	0.188	1.700	0.033	1.452	38.830	0.207	1.365	0.054
K	3338.35	0.292	− 29.640	3.846	13.912	0.151	2.177	0.036	1.424	39.713	0.207	1.164	0.049
K	3338.75	0.352	− 29.640	4.462	15.977	0.184	1.696	0.035	1.503	37.606	0.198	1.334	0.056
K	3339.35	0.151	− 29.155	1.311	5.053	0.050	0.692	0.027	0.915	47.701	0.113	0.385	0.033
K	3339.75	0.381	− 29.487	4.952	17.562	0.213	1.650	0.033	1.502	36.711	0.223	1.463	0.058
K	3340.20	0.391	− 29.537	4.903	17.081	0.204	1.945	0.032	1.464	36.201	0.209	1.467	0.063
K	3340.90	0.236	− 29.641	2.733	9.927	0.105	0.926	0.031	1.053	43.214	0.161	0.816	0.044
K	3341.38	0.263	− 29.530	3.280	11.531	0.136	2.129	0.030	1.107	40.291	0.168	0.993	0.043
K	3341.85	0.401	− 29.102	4.693	17.265	0.199	6.725	0.031	1.327	32.711	0.199	1.432	0.067
K	3342.37	0.185	− 29.643	2.216	8.785	0.087	0.868	0.030	0.972	44.246	0.148	0.661	0.039
K	3342.90	0.260	− 29.625	3.209	12.512	0.127	1.582	0.035	1.138	40.756	0.183	0.963	0.047
K	3343.35	0.142	− 29.522	1.984	7.983	0.074	0.679	0.030	0.962	44.347	0.155	0.588	0.039
K	3343.77	0.203	− 29.581	1.887	8.769	0.072	1.132	0.039	0.861	44.008	0.167	0.560	0.034
K	3344.33	0.197	− 29.376	2.395	11.541	0.099	0.874	0.040	0.916	42.693	0.234	0.705	0.036
K	3346.15	1.279	− 28.955	10.268	41.891	0.422	3.004	0.025	2.139	17.404	0.432	3.154	0.087
K	3346.68	3.654	− 28.603	13.236	60.045	0.508	4.456	0.019	1.862	1.722	0.477	4.176	0.104
K	3347.36	3.598	− 28.558	15.973	56.365	0.647	4.740	0.021	2.371	2.762	0.578	5.030	0.156
K	3348.12	1.964	− 28.155	12.275	42.156	0.484	4.278	0.030	2.228	16.407	0.464	3.538	0.113
K	3348.92	1.755	− 28.189	9.915	36.013	0.396	3.515	0.031	2.082	21.335	0.425	2.846	0.101
K	3349.44	1.603	− 28.243	10.096	37.117	0.406	3.372	0.035	2.125	21.834	0.462	2.878	0.117
K	3350.12	1.553	− 28.166	9.882	35.543	0.398	3.218	0.035	2.110	22.049	0.454	2.860	0.110
K	3350.94	3.912	− 27.599	13.274	53.249	0.617	6.108	0.021	2.088	3.968	0.511	4.160	0.236
K	3351.35	4.362	− 27.623	12.076	54.147	0.513	4.139	0.026	1.926	7.144	0.473	3.600	0.122
K	3352.00	2.346	− 29.072	9.501	67.109	0.409	3.159	0.020	1.614	4.602	0.532	2.616	0.115
K	3352.51	4.101	− 27.572	10.173	45.704	0.396	4.010	0.041	1.959	13.982	0.479	3.034	0.093
K	3353.05	1.402	− 28.668	15.001	55.527	0.572	4.411	0.030	2.196	6.470	0.493	4.404	0.095
K	3353.82	1.185	− 28.669	14.020	54.619	0.587	4.427	0.031	2.172	7.667	0.511	4.355	0.095
K	3354.69	0.959	− 28.852	14.870	49.292	0.602	4.660	0.038	2.422	10.575	0.426	4.807	0.097
K	3355.18	0.727	− 28.949	13.782	44.937	0.533	4.304	0.036	2.337	12.049	0.395	4.395	0.078
K	3355.78	0.576	− 28.827	14.580	45.990	0.565	5.898	0.041	2.429	11.817	0.416	4.654	0.085
K	3356.39	0.886	− 28.913	15.575	49.190	0.618	4.687	0.033	2.491	9.993	0.420	4.951	0.097
K	3356.98	0.730	− 29.016	14.798	47.104	0.559	4.559	0.031	2.399	10.212	0.398	4.636	0.085
K	3357.87	0.612	− 28.957	10.559	33.802	0.411	15.784	0.045	1.817	10.912	0.319	3.340	0.081
K	3358.43	0.177	− 29.156	3.231	10.634	0.116	1.022	0.116	0.893	44.734	0.126	0.998	0.038
K	3359.13	0.581	− 28.543	15.243	47.565	0.569	5.478	0.044	2.468	10.249	0.423	4.890	0.227
WG	3243.45	2.726	− 30.058	10.060	42.620	0.470	4.700	0.040	3.600	14.090	0.230	3.640	0.120
WG	3244.15	5.751	− 29.945	9.010	39.110	0.430	4.950	0.040	2.910	15.100	0.340	3.430	0.160
WG	3244.55	6.175	− 29.210	12.450	42.700	0.540	4.290	0.030	2.000	12.070	0.450	4.590	0.310
WG	3245.46	2.834	− 29.256	4.860	18.230	0.200	2.200	0.040	1.130	38.000	0.230	1.900	0.160
WG	3246.38	4.451	− 28.966	6.420	24.760	0.270	3.610	0.040	1.540	29.630	0.200	2.690	0.130
WG	3247.40	4.255	− 29.230	10.030	40.510	0.390	4.380	0.030	1.710	17.280	0.390	3.620	0.140
WG	3248.15	5.500	− 29.287	12.830	47.370	0.480	4.540	0.030	1.860	8.790	0.350	4.730	0.150
WG	3248.83	1.290	− 29.390	1.830	17.430	0.070	0.980	0.050	1.090	42.320	0.080	0.750	0.060
WG	3249.43	3.869	− 28.967	7.980	34.880	0.320	3.200	0.030	1.630	23.810	0.240	3.180	0.170
WG	3250.28	1.072	− 28.883	10.790	41.030	0.380	2.890	0.040	2.040	19.190	0.250	4.060	0.130
WG	3250.78	2.244	− 29.388	13.630	47.040	0.510	4.200	0.030	2.500	10.370	0.420	4.960	0.210
WG	3251.62	−	−	12.100	45.490	0.460	3.330	0.030	2.050	13.640	0.410	4.600	0.280
WG	3252.15	2.636	− 29.082	11.570	41.550	0.430	3.740	0.030	1.940	16.440	0.350	4.160	0.240
WG	3253.10	0.927	− 29.151	1.960	7.970	0.080	1.230	0.050	1.130	48.140	0.090	0.750	0.110
WG	3253.53	1.633	− 29.093	15.950	54.770	0.480	3.220	0.020	1.980	5.900	0.320	5.530	0.110
WG	3254.05	5.742	− 29.019	4.830	32.790	0.200	2.700	0.030	1.080	27.380	0.130	2.150	0.130
WG	3254.56	5.874	− 28.779	7.550	48.230	0.330	3.020	0.030	1.470	14.390	0.180	3.290	0.180
WG	3255.74	2.622	− 29.279	8.940	50.510	0.350	2.810	0.020	1.440	13.680	0.200	3.690	0.190
WG	3256.14	3.347	− 29.122	7.980	53.280	0.310	3.440	0.020	1.330	11.860	0.190	3.310	0.190
WG	3256.53	5.327	− 29.238	1.770	25.780	0.080	1.350	0.030	0.900	38.510	0.070	0.800	0.060
WG	3257.10	1.002	− 29.059	12.610	46.350	0.480	4.920	0.020	1.710	9.050	0.240	4.740	0.230
WG	3257.55	6.622	− 29.106	13.110	45.660	0.490	6.180	0.020	1.780	8.700	0.270	4.780	0.200
WG	3258.65	5.824	− 29.037	11.350	39.740	0.470	7.220	0.020	1.820	7.210	0.520	4.200	0.270
WG	3259.12	12.679	− 28.759	9.200	42.520	0.480	4.810	0.030	3.530	13.370	0.430	3.660	0.120
WG	3260.38	5.110	− 28.681	9.380	45.540	0.360	2.350	0.030	1.660	17.570	0.250	3.690	0.060
WG	3260.90	1.610	− 29.230	12.540	56.110	0.430	2.570	0.020	1.420	8.280	0.260	4.730	0.100
WG	3261.40	2.688	− 29.378	6.040	42.330	0.250	3.550	0.030	1.370	18.630	0.140	2.690	0.130
WG	3262.03	6.314	− 29.515	6.230	42.260	0.270	2.010	0.030	0.870	22.130	0.160	2.630	0.090
WG	3262.68	–	–	10.800	49.860	0.450	2.280	0.020	1.240	12.860	0.260	4.290	0.120
WG	3263.00	3.527	− 29.691	3.290	39.170	0.130	2.020	0.030	0.940	27.730	0.120	1.400	0.060
WG	3264.60	1.891	− 29.842	6.660	52.700	0.260	2.750	0.020	1.320	14.050	0.170	2.790	0.120
WG	3265.15	4.145	− 29.847	9.190	58.390	0.330	2.940	0.020	1.180	8.520	0.220	3.760	0.120
WG	3265.67	3.849	− 29.716	3.800	35.470	0.170	1.040	0.030	1.010	30.070	0.140	1.670	0.070
WG	3266.10	1.153	− 29.683	5.510	30.170	0.230	8.080	0.040	1.630	22.860	0.130	2.360	0.070
WG	3266.17	3.047	− 29.402	6.760	35.080	0.280	2.000	0.030	1.250	26.510	0.190	2.770	0.170
WG	3266.62	0.174	− 29.471	1.370	7.320	0.050	0.630	0.110	0.540	50.540	0.050	0.530	0.050
WG	3267.1	5.100	− 29.415	13.820	49.190	0.520	4.070	0.020	1.610	7.790	0.280	5.270	0.170
WG	3267.75	2.551	− 29.299	7.010	39.200	0.260	2.880	0.030	1.140	23.400	0.180	2.650	0.090
WG	3268.80	3.091	− 29.372	9.020	53.150	0.320	3.130	0.020	1.180	13.140	0.220	3.460	0.110
WG	3269.28	2.685	− 29.221	9.040	55.680	0.330	2.860	0.020	1.370	11.580	0.220	3.490	0.090
WG	3270.07	4.735	− 27.851	9.490	42.550	0.330	3.810	0.030	1.640	16.310	0.200	3.750	0.200
WG	3271.24	5.944	− 27.791	9.060	51.800	0.340	3.260	0.020	1.340	11.040	0.200	3.560	0.190
WG	3271.75	3.338	− 29.586	9.380	44.310	0.410	1.880	0.020	1.210	18.000	0.210	3.910	0.130
WG	3272.55	7.379	− 27.836	5.460	58.330	0.220	3.390	0.020	1.100	9.970	0.150	2.160	0.200
WG	3273.10	2.846	− 27.786	5.620	23.240	0.260	3.210	0.050	2.090	33.100	0.120	2.180	0.080
WG	3273.75	13.219	− 27.360	8.730	42.620	0.360	8.590	0.020	1.280	9.080	0.150	3.450	0.110
WG	3274.48	0.682	− 27.100	13.400	41.650	0.530	3.500	0.060	1.900	16.560	0.330	4.460	0.070
ESB	2189.89	–	–	8.180	31.320	0.380	3.480	0.030	2.400	23.040	0.120	3.970	0.150
ESB	2190.52	2.515	− 30.187	4.270	22.760	0.230	1.660	0.030	2.740	33.540	0.080	2.220	0.060
ESB	2191.12	0.170	− 28.514	0.240	6.600	0.020	0.790	0.030	1.150	52.000	0.020	0.150	0.050
ESB	2191.53	4.256	− 30.064	6.560	24.600	0.320	2.400	0.030	1.980	29.740	0.110	3.090	0.150
ESB	2192.01	0.168	− 29.100	2.780	7.450	0.100	0.840	0.020	1.090	47.290	0.100	0.960	0.030
ESB	2192.43	9.761	− 30.248	8.360	30.070	0.410	3.520	0.020	2.290	19.650	0.130	3.940	0.280
ESB	2192.78	1.472	− 29.253	1.890	8.230	0.100	0.900	0.030	0.940	47.290	0.050	0.940	0.120
ESB	2193.12	5.739	− 29.832	4.990	17.310	0.220	1.920	0.030	1.050	35.370	0.100	2.550	0.340
ESB	2193.75	7.671	− 28.800	7.190	24.050	0.310	2.210	0.030	1.340	27.550	0.110	3.480	0.290
ESB	2194.52	0.602	− 28.247	2.170	9.130	0.120	1.140	0.030	1.890	46.050	0.050	1.130	0.110
ESB	2194.95	0.356	− 27.974	1.310	5.110	0.080	0.410	0.040	0.830	50.710	0.040	0.600	0.040
ESB	2195.23	1.141	− 28.604	5.030	21.330	0.290	2.310	0.040	5.700	30.780	0.100	2.370	0.080
ESB	2196.28	0.204	− 28.466	0.550	2.780	0.030	2.500	0.050	3.480	49.350	0.050	0.290	0.520
ESB	2196.84	0.052	− 29.454	1.160	4.130	0.050	0.960	0.090	1.370	51.050	0.060	0.440	0.030
ESB	2197.3	0.223	− 29.129	7.310	19.340	0.300	2.420	0.040	1.960	34.730	0.190	2.570	0.050
ESB	2197.8	0.136	− 28.949	3.340	9.730	0.140	1.150	0.030	1.180	45.620	0.100	1.230	0.040
ESB	2198.93	0.153	− 28.553	4.230	12.370	0.170	1.410	0.030	1.460	42.610	0.130	1.640	0.040
ESB	2199.3	0.247	− 28.824	4.150	11.410	0.170	1.270	0.030	1.320	43.730	0.140	1.500	0.060
ESB	2199.54	0.233	− 28.172	8.320	21.660	0.320	2.420	0.030	2.010	32.990	0.240	3.000	0.050
ESB	2200.04	0.191	− 29.108	2.610	7.650	0.090	0.910	0.030	1.090	47.510	0.090	0.970	0.030
ESB	2200.7	0.145	− 28.556	4.530	12.570	0.170	1.420	0.030	1.420	42.890	0.150	1.640	0.030
ESB	2201.14	0.223	− 28.391	7.250	19.110	0.280	2.390	0.030	2.190	35.560	0.180	2.640	0.060
ESB	2201.55	0.112	− 28.526	3.690	10.560	0.150	1.220	0.030	1.290	44.710	0.150	1.380	0.020
ESB	2202.04	0.328	− 28.834	5.170	14.690	0.200	1.630	0.030	1.690	40.600	0.170	1.960	0.020
ESB	2202.53	0.289	− 28.667	1.370	5.190	0.060	0.680	0.040	0.980	50.320	0.060	0.610	0.040
ESB	2202.98	0.275	− 29.081	2.690	8.390	0.120	1.000	0.030	1.250	47.130	0.120	1.140	0.060
ESB	2203.33	0.168	− 28.531	3.250	9.570	0.130	1.060	0.030	1.410	45.360	0.140	1.270	0.040
ESB	2203.95	0.177	− 28.624	3.940	11.280	0.160	1.220	0.030	1.430	43.770	0.130	1.560	0.040
ESB	2204.45	0.188	− 28.596	1.050	4.230	0.050	0.580	0.050	0.910	51.260	0.060	0.490	0.030
ESB	2205.01	0.242	− 28.959	1.260	4.000	0.050	0.530	0.050	0.830	51.150	0.070	0.510	0.040
ESB	2205.37	0.189	− 27.903	1.550	4.570	0.060	0.570	0.030	0.870	50.600	0.070	0.610	0.040
ESB	2206	0.245	− 29.057	2.300	6.580	0.090	0.730	0.030	1.110	48.620	0.080	0.900	0.050
ESB	2206.37	0.215	− 28.948	1.230	4.550	0.060	0.610	0.030	0.920	51.010	0.060	0.550	0.030
ESB	2207.15	0.104	− 28.717	0.280	1.290	0.020	0.360	0.040	0.620	54.830	0.040	0.120	0.030
ESB	2207.7	–	–	0.570	3.030	0.030	0.420	0.030	0.720	54.220	0.040	0.290	0.030
ESB	2207.7	0.284	− 29.359	4.410	12.540	0.180	1.470	0.040	1.550	42.990	0.130	1.730	0.030
ESB	2208.63	0.307	− 28.805	0.630	2.580	0.040	0.430	0.030	0.760	53.800	0.040	0.320	0.030
ESB	2209.17	0.255	− 29.285	0.700	2.460	0.030	0.660	0.050	0.650	53.270	0.050	0.340	0.130
ESB	2209.7	0.242	− 28.912	1.030	4.130	0.040	0.560	0.040	0.860	51.470	0.050	0.490	0.040
ESB	2210.1	0.250	− 29.077	0.800	2.870	0.030	0.370	0.030	0.710	52.930	0.040	0.350	0.030
ESB	2210.65	0.168	− 29.377	0.840	2.930	0.040	0.430	0.030	0.640	52.760	0.050	0.370	0.120
ESB	2211	0.334	− 28.885	1.620	5.130	0.090	0.690	0.040	0.790	50.530	0.060	0.680	0.050
ESB	2211.5	0.235	− 28.715	1.030	4.210	0.050	3.230	0.040	0.790	48.470	0.050	0.510	0.060
ESB	2212.1	0.152	− 28.473	0.740	3.210	0.040	0.420	0.040	0.700	52.870	0.050	0.370	0.040
ESB	2212.43	–	–	1.770	4.840	0.070	0.640	0.030	0.700	50.360	0.060	0.670	0.060
ESB	2213.05	0.312	− 28.172	2.540	7.920	0.110	0.820	0.030	1.020	47.510	0.090	1.010	0.070
ESB	2213.52	0.200	− 28.455	1.620	5.350	0.080	0.500	0.030	0.770	50.410	0.070	0.650	0.040
ESB	2213.95	0.239	− 28.225	9.820	25.260	0.360	2.310	0.040	1.750	30.440	0.270	3.350	0.060
ESB	2214.4	0.178	− 28.657	9.950	27.210	0.400	2.980	0.040	2.500	26.950	0.240	3.530	0.050
ESB	2214.96	0.462	− 28.423	2.950	9.660	0.100	0.710	0.040	0.970	46.470	0.080	1.180	0.030
ESB	2215.85	1.498	− 28.865	3.570	15.870	0.180	1.180	0.030	1.360	40.360	0.070	1.890	0.080
ESB	2216.13	0.221	− 28.505	0.500	2.620	0.040	0.220	0.030	0.520	53.390	0.030	0.270	0.060
ESB	2216.79	0.825	− 28.711	3.050	14.640	0.180	1.060	0.030	2.800	40.350	0.070	1.570	0.060
ESB	2217.78	0.550	− 29.032	0.550	3.890	0.030	1.390	0.060	0.560	52.700	0.030	0.300	0.040
ESB	2218.7	1.384	− 29.632	1.030	5.160	0.050	0.580	0.030	0.590	50.560	0.040	0.560	0.140
ESB	2218.85	0.250	− 28.448	0.410	2.180	0.020	2.430	0.090	0.850	53.720	0.030	0.230	0.140
ESB	2219.2	1.010	− 28.972	0.750	3.370	0.040	0.380	0.030	0.550	52.070	0.040	0.410	0.120
ESB	2219.88	3.057	− 29.093	2.480	7.630	0.120	1.960	0.030	0.740	45.880	0.050	1.110	0.110
ESB	2220.18	0.122	− 26.122	0.560	3.120	0.030	2.240	0.060	1.610	53.030	0.040	0.300	0.110
ESB	2220.68	0.172	− 28.424	0.950	3.650	0.050	2.660	0.050	1.530	51.690	0.050	0.390	0.050
ESB	2221.17	0.258	− 28.718	3.480	9.540	0.120	0.820	0.020	1.080	46.100	0.130	1.160	0.030
ESB	2222.46	0.248	− 28.791	2.140	6.460	0.070	0.570	0.020	1.000	48.880	0.080	0.730	0.020
ESB	2222.47	0.244	− 28.615	2.970	8.080	0.100	1.000	0.020	1.020	47.540	0.100	0.970	0.020
ESB	2223	0.288	− 28.930	1.930	6.220	0.070	0.700	0.020	1.120	49.290	0.080	0.690	0.030
ESB	2223.52	0.221	− 28.838	1.940	5.830	0.070	0.540	0.020	1.020	49.630	0.070	0.670	0.020
ESB	2223.94	0.369	− 29.025	3.120	8.840	0.120	0.960	0.020	1.370	46.590	0.090	1.080	0.020
ESB	2224.6	0.517	− 29.049	3.930	11.680	0.150	1.050	0.030	1.670	43.260	0.120	1.360	0.040
ESB	2225.15	0.357	− 28.931	4.450	13.170	0.180	1.280	0.030	2.010	41.740	0.140	1.540	0.040
ESB	2225.62	0.455	− 28.866	4.380	12.610	0.150	1.010	0.030	1.450	42.770	0.120	1.470	0.030
ESB	2226.07	–	–	6.050	17.230	0.230	1.520	0.030	2.300	37.510	0.170	2.090	0.040
ESB	2226.48	0.383	− 28.799	10.330	29.180	0.420	2.830	0.040	3.940	24.470	0.320	3.570	0.060
ESB	2227.06	0.369	− 28.950	4.610	13.710	0.160	1.130	0.040	1.500	41.810	0.130	1.580	0.040
ESB	2227.52	0.272	− 28.920	7.290	21.450	0.310	1.840	0.040	2.890	32.800	0.190	2.570	0.060
ESB	2228.02	0.597	− 27.502	11.390	35.010	0.550	3.350	0.040	6.270	16.570	0.420	4.300	0.070
ESB	2228.28	0.278	− 28.605	10.200	31.570	0.450	2.710	0.040	5.160	21.190	0.320	4.030	0.070
ESB	2228.81	0.281	− 28.056	7.510	24.830	0.330	2.430	0.040	3.730	29.710	0.200	3.090	0.060
ESB	2229.23	5.707	− 28.873	2.160	11.400	0.110	1.930	0.030	0.930	42.570	0.060	1.270	0.220
ESB	2229.6	5.641	− 28.681	7.040	27.470	0.350	3.340	0.030	2.930	25.230	0.130	3.740	0.120
ESB	2230.2	1.439	− 28.365	5.100	24.890	0.270	2.080	0.030	4.480	30.090	0.150	2.520	0.070
ESB	2230.5	1.455	− 28.134	5.710	27.540	0.330	2.570	0.040	7.590	24.230	0.150	2.860	0.070
ESB	2231.1	1.990	− 28.484	5.340	26.320	0.310	2.620	0.030	5.880	27.020	0.130	2.750	0.060
ESB	2231.49	3.430	− 28.248	5.100	19.930	0.270	2.990	0.030	2.920	30.640	0.140	2.450	0.050
ESB	2231.77	4.391	− 28.729	7.220	26.170	0.370	3.560	0.030	3.010	23.550	0.160	3.350	0.070
ESB	2232.15	0.071	− 28.511	1.100	5.870	0.070	0.310	0.020	0.690	50.850	0.050	0.580	0.020
ESB	2232.65	0.167	− 27.660	0.550	5.720	0.030	0.550	0.030	0.930	51.200	0.030	0.330	0.050
ESB	2232.9	3.131	− 29.587	6.560	26.270	0.330	1.700	0.030	2.860	29.120	0.120	3.510	0.120
ESB	2233.19	5.650	− 29.350	4.250	20.480	0.200	2.330	0.020	1.010	35.220	0.090	2.430	0.090
ESB	2233.98	–	–	2.820	10.830	0.140	1.190	0.020	1.170	44.150	0.070	1.450	0.050
ESB	2234.55	0.813	− 29.286	1.100	3.840	0.060	0.420	0.030	0.750	51.390	0.050	0.510	0.060
ESB	2235.02	3.063	− 29.450	4.360	14.270	0.210	1.830	0.020	1.280	40.470	0.130	1.980	0.040
ESB	2235.36	0.112	− 28.746	1.530	5.240	0.080	0.560	0.030	0.800	50.580	0.060	0.720	0.030
ESB	2235.98	0.120	− 28.712	0.730	2.870	0.030	0.260	0.030	0.610	52.760	0.050	0.320	0.020
ESB	2236.33	0.381	− 29.248	2.700	7.920	0.120	0.600	0.020	0.850	47.570	0.090	1.130	0.050
ESB	2237	0.347	− 29.114	1.800	5.600	0.080	0.600	0.020	1.030	49.450	0.080	0.760	0.050
ESB	2237.59	1.036	− 29.221	1.630	6.380	0.090	0.730	0.030	0.740	49.690	0.060	0.910	0.060
ESB	2238.14	–	–	4.660	15.800	0.200	1.990	0.020	1.460	40.080	0.150	2.190	0.070
ESB	2238.59	0.563	− 29.196	4.620	13.540	0.180	1.050	0.020	0.840	42.600	0.150	1.860	0.030
ESB	2239.22	2.453	− 29.241	6.250	19.280	0.280	1.410	0.020	1.140	35.610	0.150	3.000	0.070
ESB	2239.59	1.381	− 27.499	2.770	9.200	0.100	0.640	0.020	0.720	46.080	0.070	1.360	0.030
ESB	2240.06	2.224	− 29.248	6.090	18.990	0.270	1.200	0.020	0.940	36.570	0.120	3.030	0.060
ESB	2240.46	0.315	− 28.104	0.820	3.000	0.050	1.450	0.020	0.690	53.040	0.030	0.420	0.070
ESB	2240.88	0.159	− 27.398	0.440	1.980	0.020	0.350	0.020	0.900	53.120	0.030	0.230	0.050
ESB	2241.27	–	–	1.790	6.440	0.090	0.760	0.020	1.140	48.930	0.060	0.870	0.050
ESB	2241.75	0.537	− 27.769	2.030	6.510	0.100	0.490	0.020	1.080	48.680	0.050	0.980	0.020
ESB	2242.22	0.411	− 27.158	0.380	1.530	0.020	0.280	0.050	0.600	53.930	0.030	0.170	0.040
ESB	2242.63	0.130	− 25.946	0.640	2.840	0.030	0.710	0.060	0.570	53.620	0.040	0.290	0.030
ESB	2243.22	19.785	− 27.382	5.290	15.810	0.280	7.680	0.030	1.340	19.380	0.090	2.350	0.240
ESB	2244	0.335	− 29.557	1.220	4.820	0.060	0.480	0.080	0.860	50.910	0.040	0.500	0.030
ESB	2244.26	0.350	− 27.857	3.170	11.740	0.170	4.680	0.050	2.450	38.350	0.070	1.460	0.030
ESB	2244.53	–	–	4.300	13.620	0.190	1.310	0.040	1.610	41.710	0.080	1.870	0.050
ESB	2244.88	1.074	− 28.035	5.340	16.800	0.240	1.380	0.040	1.680	38.580	0.110	2.300	0.050
ESB	2245.3	0.570	− 28.491	2.000	7.290	0.070	0.640	0.040	0.860	48.370	0.050	0.890	0.020
ESB	2245.88	0.567	− 29.079	3.130	9.670	0.110	0.670	0.030	0.910	46.340	0.080	1.320	0.030

Values of Si_EX_^20^ that were calculated using Eq. (1) are plotted versus depth to show the spatial distribution of Si_EX_ in the three Duvernay wells (Fig. 2). Values below zero indicate that all of the SiO_2_ in the sample is associated with Al_2_O_3_, which is used here as a proxy for shale content. Samples from the ESB core, which contains samples from the Duvernay, shows only minor Si_EX_, with an average calculated value of -0.45%. Average values of Si_EX_ in the Kaybob and WG wells are 12.51% and 6.00%, respectively, and average values for the Duvernay in Kaybob and WG are 17.37% and 10.94%, respectively, with Si_EX_ absent in the middle carbonate in the Kaybob well. A sample from the Kaybob well shows the highest value of Si_EX_ 50.37% and the maximum value in the WG well is 39.01%. δ^13^C_(org)_ ranges from − 29.835 to − 25.709‰, − 30.057 to − 27.099‰, and − 30.248 to − 25.945‰ in the Kaybob, WG, and ESB wells, respectively.

Figure 2 also displays hyperspectral images produced for opal-A content, Al_2_O_3_, and TOC. Al_2_O_3_ is a clay proxy and shows an overall negative correlation to elevated Si_EX._ Intervals of elevated TOC correspond to increased Si_EX_ in the Kaybob well, but in the other two wells there appears to be minimal correlation between these two components.

**Figure 3:**
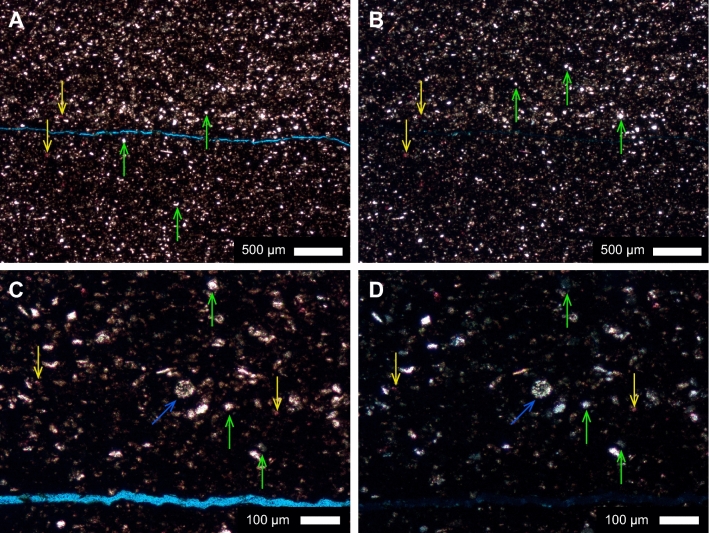
Thin section photos from the Kaybob well (04-19-064-22W4). All sections were polished, impregnated with blue epoxy to highlight porosity, and half stained with Alizarin red to detect calcite and aragonite content. (**A**) Depth 3309 m (upper Duvernay), plane polarized light, SiO_2_ (yellow arrows, down), and CaCO_3_ (green arrows, pointing up). (**B**) Same as A in crossed polarized light. (**C**) Depth 3309 m (upper Duvernay), plane polarized light, SiO2 (yellow arrows, down), CaCO3 (green arrows, up), possible radiolarian (blue arrow, diagonal). (**D**) Same as (**C**) in crossed polarized light.

Thin sections from the intervals of enriched amorphous SiO_2_ detected by the hyperspectral analysis, contained evidence of biogenic SiO_2_. SiO_2_ was detected as large (~ 30 μm diameter; Fig. 3) rounded to sub-rounded, spherical to sub-spherical particles; some with spiky projections.

**Figure 4:**
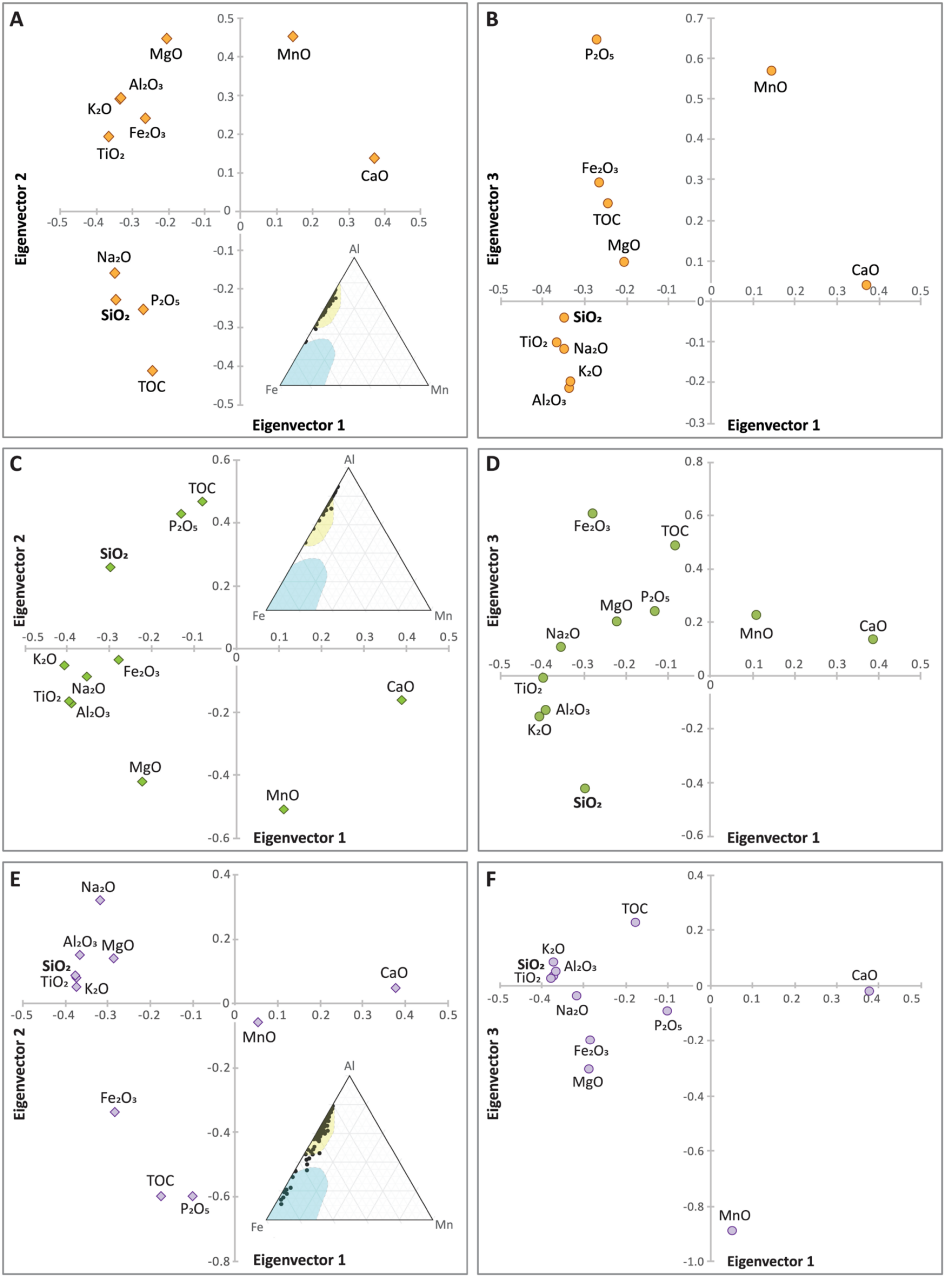
PCA analysis of Duvernay Formation oxides in (**A**,**B**) Kaybob, (**C**,**D**) Willesden Green, (**E**,**F**). East Shale Basin. Al–Fe–Mn ternary plot inserts in (**A**–**C**) determine whether element chemistry infers a possible hydrothermal source (blue) or non-hydrothermal (yellow)(Adachi et al^42^.).

Principle component analysis (PCA) used to analyze reported oxide values (Fig. 4) and TOC reveal that in the Kaybob well, SiO_2_ correlates with P_2_O_5_, TOC, and Na_2_O, in e1 versus e2 eigenvectors (Fig. 4a). When a third vector (e3) is plotted against PC1, SiO_2_ correlates with clay indicator oxides (e.g. Al_2_O_3_, TiO_2_, K_2_O; Fig. 4b). In the WG e1 versus e2 plot SiO_2_ correlates to P_2_O_5_ and TOC and e1 versus e3 plots show SiO_2_ more closely associated with the main group of clay-associated oxides (Fig. 4c,d). SiO_2_ is consistently associated with clay indicators in the ESB well (Fig. 4e,f).

Al–Fe-Mn plots for Kaybob and WG wells show samples plotting outside of the field corresponding to a hydrothermal Si source, as defined by Adachi et al.^42^. Nine ESB samples plot inside this field, and the rest in the same region as the WG and Kaybob samples (Fig. 4).

**Figure 5:**
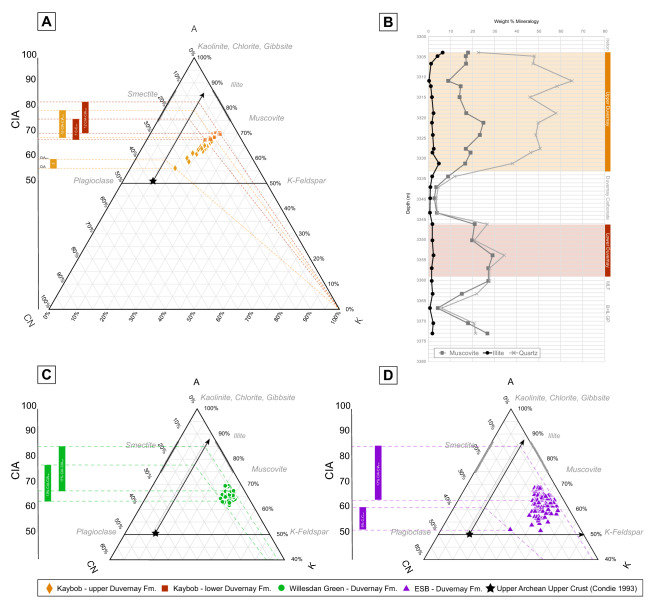
A-CN-K (A1_2_O_3_–CaO* + Na_2_O–K_2_O) plots of Kaybob well (**A**) and XRD data versus depth (**B**). The chemical alteration index (CIA) is used to find the level of diagenetic alteration the samples have experienced^44–48^. Samples are plotted back to the weathering trend along a diagenesis line (from K_2_O), to the weathering trend and over to the CIA (CIA_corr_)^46^ to correct for K-enrichment. The CIA-CIA_corr_ of the sample determines the level of K-enrichment for each sample. The highest values of CIA-CIA_corr_ are noted here for each well.

The level of diagenesis (K-metasomatism) experienced in the Duvernay in each well, as determined by CIA-CIA_corr_, ranges from 9 to 17 for ESB, 14–17 for WG, 7–12 for the lower Duvernay in Kaybob, and 3–10 for the upper Duvernay (Fig. 5).

## Discussion

### The *punctata* event in this study

The *punctata* Event (pE) was first recognized by a large positive δ^13^C excursion (up to 4.5‰) in the Frasnian *punctata* (conodont) Zone in Poland, Czech Republic, and China, but did not seem to correlate to any major sea level change or climatic fluctuation^4^. A key change was taking place in the terrestrial environment, however, the proliferation of aneurophyte and archaeopterid progymnosperm forests^1^ resulted in deeper and more complex root networks and increased pedogenesis^2,7,8^. This transition marks a significant shift in the interaction between the lithosphere and the hydrosphere. Pedogenic weathering introduced higher levels of terrestrially-derived nutrients (e.g. P) into the marine environment resulting in increased primary productivity, and oxygen stratification^8^. The burial of organic carbon associated with bottom water anoxia combined with increased upper water column productivity elevated δ^13^C ratios^50^, resulting in several positive excursions seen from the late Devonian through to the Carboniferous^1,8^.

**Figure 6:**
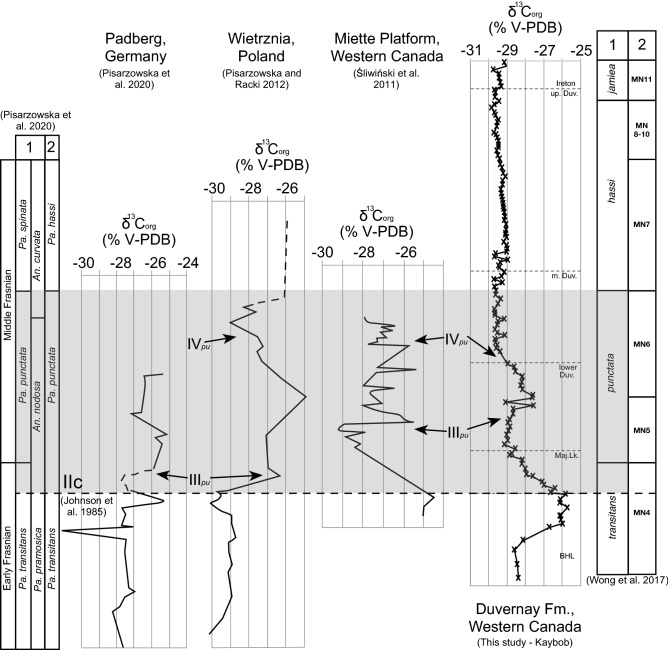
δ^13^C_(org)_ records from Padberg, Germany^12^, Wietrznia, Poland^52^, western Canada (Miette Platform)^18^, and this study of the Duvernay Formation from western Canada. These records are plotted with the biostratigraphic column from Pisarzowska et al.^12^ (1 = revised conodont zonation in Pisarzowska et al.^12^ and 2 = Racki and Bultynck^53^) and Wong et al.^31^ (1 = Ziegler and Sandberg^54^ and 2 = Montagne Noire Zone^55^).

In this study, positive δ^13^C_(org)_ isotope excursions (2.9–3.7‰) are present near the base of the Duvernay Formation (Fig. 2). The isotopic shift at the base of the Duvernay/Perdrix was first recognized in the WCSB in the subsurface by Holmden et al.^51^, and in the Rocky Mountains in Alberta by Śliwiński et al.^9^, who recognized it as the pE. Śliwiński et al.^18^ Fig. 7 reveals two δ^13^C_(org)_ excursions, one in the Maligne Formation (Majeau Lake Formation equivalent), thought to be a side effect of dolomitization, and one in the Perdrix Formation (Duvernay Formation equivalent). The positive shift in the Perdrix is Events III of the pE and the abrupt return to background values is Event IV (Fig. 6), according to comparison of recently updated Frasnian conodont stratigraphy in the WCSB^32^, and correlations between numerous locations worldwide in Pisarowska et al.^12^ In this study, the Kaybob well displays two shifts, one larger positive excursion in the Waterways Formation of the Beaverhill Lake Group through into the Majeau Lake Formation, and another in the lower Duvernay. Śliwiński et al.^18^ also records this positive shift in the Majeau Lake/Maligne interval but recommends caution in its interpretation, since this interval contains dolomitized strata. Although, dolomitization is not usually considered as likely to affect δ^13^C_(org)_ isotope data and this positive perturbation is present in the Kaybob well of this study at the same interval implying that this interval might be worth further investigation, despite its divergence from the worldwide records. The WG, Kaybob, and ESB wells each display a δ^13^C_(org)_ excursion in the lower Duvernay, of which the base, positive excursion is considered to be Event III, and the return to background values, Event IV^12^ (Fig. 6). The slight offset of these excursions is not surprising considering that the timing and rate of extensive plant colonization and development would be staggered throughout the globe^8^. The δ^13^C_(org)_ excursion in the Kaybob well, which is the most distal of the three wells included in the basin transect has the weakest Event IV δ^13^C_(org)_ perturbation (1.5‰) and the ESB well, which is the most proximal and deposited on a pre-existing carbonate shelf (Cooking Lake Formation), displays the strongest perturbation (3.7‰). This shift in the magnitude of δ^13^C_(org)_ values across the basin follows the model proposed by Śliwiński et al.^18^ that found the magnitude of the pE δ^13^C_(org)_ excursion for Event III is reduced from shelf to basin. With Event IV of the pE identified in each well, the LWIR data and Si_EX_ was compared to δ^13^C_(org)_ data to determine whether predicted increased productivity (plankton = radiolarians), correlate with the onset of forest expansion, increased soil generation and nutrient delivery, as predicted in Algeo and Scheckler^2^.

### Amorphous SiO_2_ related to the *punctata* Event

In the Kaybob core, there is a slight increase in Si_EX_ following the largest δ^13^C_(org)_ shift (3363.04 m; Event II; Fig. 2) followed by elevated Si_EX_ and opal-A that corresponds to a second, smaller δ^13^C_(org)_ shift (3352.00 m; Event III). Finally, there is a significant and sustained increase in Si_EX_ and opal-A SiO_2_ in the upper Kaybob Duvernay (3331.45 m) that does not directly correspond to a δ^13^C_(org)_ shift. In the WG core, Si_EX_ and opal-A SiO_2_ content increases when δ^13^C_(org)_ shifts to its most positive value (3274.48 m; Event III) and Si_EX_ and opal-A SiO_2_ continues to be present throughout the lower Duvernary. In the ESB well, in a position farthest from the open ocean, on the shelf, and behind several large reef platforms, there is the largest of the Event III δ^13^C_(org)_ shifts (2243.22 m) and another smaller shift up Sect. (2232.65 m) that corresponds to an increase in Si_EX_ and slightly elevated opal-A SiO_2_. Relative to the other two wells, the ESB Duvernay contains minimal Si_EX_ and opal-A SiO_2_, which is perhaps unsurprising considering it is located on the platform behind several large reef complexes. In each of the three datasets, it appears that the opal-A SiO_2_ negatively correlates with intervals of increased Al_2_O_3_, an indicator of clay input. Elevated levels of Si_EX_ and opal-A SiO_2_ may be related to δ^13^C_(org)_ shifts inferred to signal changes in the global carbon cycle linked to the pE; however, not all intervals of increased Si_EX_ and opal-A SiO_2_ are correlated to the exact onset of these excursions. There may be other sources of opal-A SiO_2_ in the basin, besides radiolaria related to the pE, therefore, it is critical to interrogate the data and ascertain whether there could also be multiple sources of amorphous SiO_2_, especially given the lack of a δ^13^C_(org)_ excursion in the upper Duvernay of the Kaybob well, where there is a significant amount of Si_EX_ identified by the LWIR as opal SiO_2_.

### Sources of amorphous SiO_2_ in the Duvernay Formation shales

The possible sources of amorphous SiO_2_ in shales or mudrocks, include: (1) a biogenic source^14,24,38^, (2) hydrothermal alteration^42^, or (3) a by-product of clay diagenesis (e.g. smectite-illite or illite to muscovite transitions)^43,56,57^.

PCA analysis (Fig. 4) of the Kaybob and WG wells show that SiO_2_ correlates with P_2_O_5_ and TOC in the e1 vs e2 (Fig. 4a,c). Higher values of P_2_O_5_ content indicate increased paleoproductivity^9,14,58,59^. The association of SiO_2_ with P_2_O_5_ and TOC may therefore suggest that Si_EX_ in WG and Kaybob is primarily biogenically sourced. This is supported by Harris et al.^38^ where Si_EX_ in the Duvernay is attributed to a biogenic source as intervals that are high in excess Si (> 5%) correlate to high TOC. Na_2_O was also associated with SiO_2_, P_2_O_5_, and TOC in this field. Na_2_O is typically considered a clay-proxy but Na_2_O values can be unreliable due to the incorporation of drilling fluids, so it is removed from our interpretation of all PCA plots in this study^41^. In PCA analysis (Fig. 4), e1 vs. e3 in the Kaybob well shows SiO_2_ associated with clay indicators (e.g. TiO_2_, Al_2_O_3_), suggesting that there is also a contribution of SiO_2_ from siliciclastics in this well, whereas the SiO_2_ in e1 vs. e3 in the WG well is not tightly clustered with clay indicators, but is also disassociated from P_2_O_5_ and TOC. This may indicate the presence of aeolian silt in the WG well since this type of silica would not necessarily be tightly correlated to clay proxies. A hydrothermal Si source can be discounted for the Kaybob and WG wells based on Al–Fe-Mn plots that reveal that these samples plot close to the Al field, indicating that they are not associated with a hydrothermal source (Fig. 4)^42^. Petrographic analysis of thin sections from the Kaybob Duvernay, specifically from the intervals (3309 m and 3350 m) of elevated Si_EX_ and shown by the hyperspectral images to contain opal-A SiO_2_, reveal several rounded particles of SiO_2_, some that display lacey or porous structures suggesting that they are radiolarians (Fig. 3c,d). All the thin section photos shown in Fig. 3 were taken in the regions that were stained with Alizarin red. The round to sub-rounded, spherical to sub-spherical particles shown in Fig. 3 (green arrows, pointing up) may also be the source of the amorphous SiO_2_ shown in the hyperspectral images (Fig. 2). Schieber^14,17^ states that identification of quartz silt in shales may in fact be algal cysts or spores that are filled with diagenetic SiO_2_, sourced from biogenic SiO_2_ (radiolaria or sponges), similar to the rounded to subrounded SiO_2_ particles in Fig. 3. In the ESB well, all PCA analysis shows SiO_2_ correlated to clay indicators, suggesting that most SiO_2_ in the ESB well is sourced from siliciclastics. However, there are nine samples that plot close to the Fe field in the Al–Fe-Mn ternary plot, which is associated with metalliferous sediments interpreted as hydrothermal precipitates (Fig. 4e)^42^. These intervals do not correlate with increased levels of Si_EX_ (Fig. 2) and overall, Si_EX_ and LWIR imagery indicates that Si_EX_ and opal-A in the ESB well is minor.

Burial clay diagenesis that may result in opal-A as a by-product of K-enrichment was evaluated as a potential source of SiO_2_ in the Duvernay wells. This was done by plotting oxide data on an A-CN-K ternary diagram^44–48^ to determine how far the samples have shifted away from the predicted A-CN parallel weathering line that predicts the loss of CaO, Na_2_O, and K_2_O, expected in chemical weathering of sediments. The distance away from this weathering trend, towards the K_2_O apex determines whether the sediments have experienced significant K-enrichment that is associated with the smectite-illite reaction, illitization or K^+^ enrichment (Fig. 5). Subsiding marine basins in lower temperature settings of 100–120 °C are likely to experience K-metasomatism that may result in smectite-illite transitions and precipitation of amorphous SiO_2_ as a by-product of the reaction^43,45,60^. From the A-CN-K plots it is evident that samples from the ESB and WG wells have experienced a similar level of K-enrichment, with CIA-CIA_corr_ values of up to 17 (Fig. 5c,d). Kaybob samples have experienced the least K-enrichment, with the upper Duvernay displaying CIA-CIA_corr_ values of only 3–10. Therefore, the least alteration was experienced in the upper Kaybob Duvernay, which also show the highest values of Si_EX_ and amorphous SiO_2_ (Fig. 2). While there is no way to definitively determine how much amorphous SiO_2_ may be contributed as a by-product of clay diagenesis, Abercrombie et al.^43^ predicts that significant volumes of SiO_2_ would not be contributed through this process. Accordingly, while all the samples from the Duvernay wells have undergone K-enrichment through clay diagenesis, which may result in some contribution of amorphous SiO_2_, it is likely that the primary source is biogenic for amorphous Kaybob and WG SiO_2_ in particular, based on the association of SiO_2_ with P_2_O_5_ and TOC in the PCA analysis (Fig. 4).

### Implications for *punctata* models, δ^13^C excursions and silica cycling

In past studies of the pE, like many other major shifts in climate and the carbon cycle, δ^13^C_(org)_ or δ^13^C_(carb)_ excursions are used to pinpoint the onset of a given event (e.g. large igneous province eruptions). During these shifts, certain associated geochemical signals are expected in the rock record that we use as proxy for understanding the causal events that led to changes to the atmosphere, biosphere, or hydrosphere. Pisarowska et al.^12^ reviews possible causes for the pE excursion and points out that the IIc^61^ flooding surface corresponds to the onset of the pE in several of the locations it is recognized in and intensified water exchange between epeiric seas or and the open ocean coupled with the changes in the terrestrial environment are the most likely cause. The Alamo impact in southern Nevada^62^ and potential volcanic eruptions^63,64^, have also been cited as possible explanations for the excursion but Pisarowska et al.^12^ points out that the onset of these events does not correspond to the onset of the pE and major volcanic eruption would be more likely to result in a negative excursion, as seen at the Permian–Triassic extinction interval. In the WCSB, reef building continues through this interval, implying that this marine life persists through this interval at this location.

With the cause of the pE considered and attributed partially to the development of deeper root networks and thicker soil horizons during the Middle to Late Devonian, it is likely that riverine discharge carrying increased concentrations of liberated P^8^ would increase along with planktonic biomass. With increased productivity and upwelling predicted through models and use of proxies^2,9^, an increase in biogenic SiO_2_ reflecting the presence of opal-A radiolarian tests at the onset of the pE may be expected. All three wells, even the ESB well display increases in opal-A at or shortly after the onset of pE Event III and based on the PCA, Al–Fe–Mn, and A-CN-K analysis presented here, the source is likely biogenic. However, the concentrated opal-A and TOC present in the Kaybob upper Duvernay, which has the lowest overall degree of K-enrichment and no evidence of hydrothermal influence, does not correspond to a δ^13^C_(org)_ excursion, despite satisfying criteria normally used to explain positive δ^13^C shifts. The significant increases in biogenic SiO_2_ and TOC have been attributed to upwelling during a second-order sea-level rise, unrelated to the pE^38^. However, increased concentrations of biogenic Si and TOC are not observed in the WG well. The distribution of opal-A in the Kaybob well agrees with established models of Duvernay deposition whereby continentally derived material is deposited in east–west clinoforms^34^. In the WG basal Duvernay there is a slight increase in biogenic Si coupled with weathered clay, and possible quartz silts (WG PCA e1 vs. e3). With westward-thinning clinoforms, the Kaybob well, which is closest to the open ocean, is more favourable for deposition through suspension, favouring preservation of siliceous skeletal material. Overall, the Kaybob well does contain less clay and more opal-A SiO_2_ than the ESB well. The conundrum illustrated in this study is that the δ^13^C_(org)_ excursions signalling the pE do not necessarily correspond to the most geochemically and mineralogically distinct interval that would seem to fit the predicted response of increased productivity and preservation of organic matter (e.g. high concentration of biogenic Si and TOC in the upper Duvernay in Kaybob). The expected response of increased productivity and preserved organic matter may be diluted in the basal Duvernay at the pE due to the accompanying increase of terrestrially-derived sediments associated with deeper root networks developed during the pE. Offsets between major climate shifts signalled by δ^13^C and the response recorded in sedimentary strata have been identified in other studies of the *punctata* Event^8^ and intervals of other significant climatic events or potential biodiversity gaps (e.g. Cambrian SPICE Event^64^; Paleocene-Eocene^65^). One thing is certain, many different methods of investigation should be used when interpreting δ^13^C excursions and the onset of significant climatic shifts, versus the changes (e.g. increased productivity) that are recorded in the rock record. The links between these are still not well understood. As observed in this study and Śliwiński et al.^18^, the magnitude of δ^13^C excursions also varies, depending on the position in the basin. The effects of major shifts in δ^13^C records (e.g., changes in productivity, increased TOC, etc.) are also variable in ocean basins and their position in the sedimentary record. Given the importance of shifts in weathering, productivity, and the biogeochemical Si cycle around major climate shifts and extinctions, separation of amorphous versus crystalline SiO_2_ is critical to future studies of these events^16,27^. The use of LWIR in this study is the first attempt at mapping amorphous and most likely biogenic SiO_2_ in an ancient basin. Further studies using this technique may help us understand biogeochemical silica cycling and detect significant shifts in this cycle through geological time. Current methods of differentiating SiO_2_ polymorphs have many limitations^27^. Early plant colonization of landscapes in the Middle to Late Devonian increased delivery of P to the marine environment, likely increasing productivity in the upper water column and localized bottom water anoxia, but with eventual stabilization of P liberation as the vegetation becomes established, it is unknown whether these shifts cause sustained basin-wide changes in the marine environment^8^. Mapping intervals of increased productivity (biogenic SiO_2_) on a basin scale will aid our understanding of terrestrial-marine teleconnections during periods of significant climatic shifts.

## Conclusions

This study examines the distribution of amorphous SiO_2_ in the Duvernay Formation in three wells across a basin transect to determine whether an increase in productivity (e.g. radiolarians) is associated with the emergence of deeper root networks and thicker soil horizons contributing to terrestrially-derived nutrients in the oceans during the *punctata* Event (pE). Previous investigation of modern and ancient records for intervals of amorphous SiO_2_ that may be attributed to biogenic sources have relied on geochemical proxies or separated aliquots that have not captured all of the amorphous SiO_2_^27^. LWIR hyperspectral imaging allows us to detect opal-A SiO_2_ on a macroscale, as a continuous dataset, using a non-destructive instrument. This ability to quickly and efficiently detect intervals of opal-A SiO_2_ means that we may start to map these intervals on a basin scale to improve paleogeographic reconstruction and map past oceanic circulation. Opal-A SiO_2_ identified by LWIR imaging was determined to have two sources: 1) a by-product of clay diagenesis identified using an A-CN-K plot, and 2) biogenic silica, which is the primary source of SiO_2_ in two of the three wells. In the case of the Duvernay in the Western Canada Sedimentary Basin, the effect of the pE resulted in increased paleoproductivity in two areas of Duvernay deposition basinward of a large barrier reef. The third area represented by the ESB well, which is located on the carbonate platform and behind several large elongated barrier reefs, shows only minor amorphous SiO_2_. While the A-CN-K plot suggests there may have been a minor contribution of Si_EX_ or opal-A SiO_2_ from clay diagenesis, the basinward increase in Si_EX_ and opal-A SiO_2_, correlation of SiO_2_ to P_2_O_5_, TOC, and the δ^13^C_(org)_ shift associated with the pE, and detection of possible radiolarian tests or SiO_2_-filled algal cysts all point to a dominantly biogenic SiO_2_ source in the Duvernay. This finding supports theories that oceans experienced an increase in productivity as the world’s forests expanded and developed deeper root networks which increased soil genesis and delivery of terrestrially-derived nutrients into the marine realm. However, the highest concentration of Si_EX_, biogenic opal-A SiO_2_ and TOC is in the upper Duvernay of the most distal well and does not correspond to a δ^13^C_(org)_ excursion. This study demonstrates the need for more studies that examine the correlation of δ^13^C excursions and reactions recorded in the sedimentary record and the need for differentiation of amorphous from crystalline SiO_2_ so that we may better understand global silica cycling through terrestrial-marine teleconnections.

## Data Availability

The datasets used and/or analysed during the current study available from the corresponding author on reasonable request.
